# Differences in the urinary metabolome and proteome between wet and dry nights in children with monosymptomatic nocturnal enuresis and nocturnal polyuria

**DOI:** 10.1007/s00467-023-05963-5

**Published:** 2023-05-04

**Authors:** Cecilie Siggaard Jørgensen, Konstantinos Kamperis, Jane Hagelskjær Knudsen, Margrethe Kjeldsen, Jane Hvarregaard Christensen, Luise Borch, Søren Rittig, Johan Palmfeldt

**Affiliations:** 1 Department of Pediatrics and Adolescent Medicine, Aarhus University Hospital, Palle Juul-Jensens Boulevard 99, DK-8200 Aarhus, Denmark; 2 Department of Clinical Medicine, Aarhus University, Aarhus, Denmark; 3 Department of Clinical Medicine—Research Unit for Molecular Medicine, Aarhus University, Aarhus, Denmark; 4 Department of Biomedicine, Aarhus University, Aarhus, Denmark; 5 Department of Paediatrics and Adolescent Medicine, Gødstrup Hospital, Herning, Denmark; 6 NIDO | Centre for Research and Education, Gødstrup Hospital, Herning, Denmark

**Keywords:** Nocturnal enuresis, Polyuria, Metabolomics, Proteomics, Oxidative stress

## Abstract

**Background:**

Nocturnal enuresis (NE) is a common disease with multiple pathogenic mechanisms. This study aimed to compare levels of metabolites and proteins between wet and dry nights in urine samples from children with monosymptomatic NE (MNE).

**Methods:**

Ten boys with MNE and nocturnal polyuria (age: 7.6 ± 1.3 years) collected their total nighttime urine production during a wet and a dry night. Untargeted metabolomics and proteomics were performed on the urine samples by liquid chromatography coupled with high-mass accuracy tandem mass spectrometry (LC-MS/MS).

**Results:**

On wet nights, we found reduced urine osmolality (*P* = 0.025) and increased excretion of urinary potassium and sodium by a factor of, respectively, 2.1 (*P =* 0.038) and 1.9 (*P* = 0.19) compared with dry nights. LC-MS identified 59 metabolites and 84 proteins with significantly different levels between wet and dry nights (fold change (FC) < 0.67 or > 1.5, *P* < 0.05). Some compounds were validated by different methodologies. During wet nights, levels of compounds related to oxidative stress and blood pressure, including adrenalin, were increased. We found reduced levels of aquaporin-2 on wet nights. The FCs in the 59 metabolites were positively correlated to the FCs in the same metabolites identified in urine samples obtained during the evening preceding wet and dry nights.

**Conclusions:**

Oxidative stress, which in the literature has been associated with nocturia and disturbances in sleep, might be increased during wet nights in children with MNE. We further found evidence of increased sympathetic activity. The mechanisms related to having wet nights in children with MNE seem complex, and both free water and solute handling appear to be important.

**Graphical abstract:**

**Figure:**
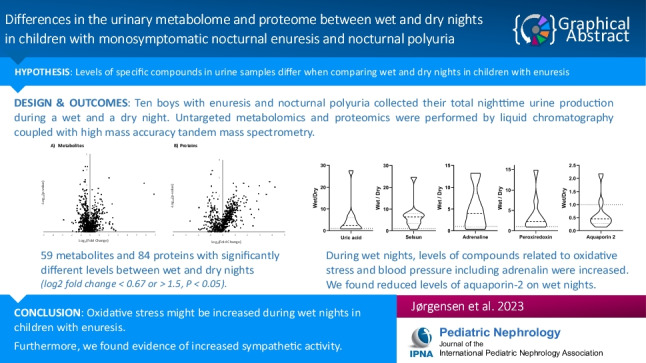
A higher resolution version of the Graphical abstract is available as Supplementary information

**Supplementary Information:**

The online version contains supplementary material available at 10.1007/s00467-023-05963-5.

## Introduction

Involuntary voiding during sleep, NE, is a common condition affecting 10–16% of all seven-year-old children [1, 2]. For many children, NE has a considerable negative impact on general well-being [3]. It is known that NE is a multifactorial disease with different pathogenic mechanisms, including an abnormal bladder reservoir function during nighttime [4, 5], nocturnal polyuria [6, 7], and inability to wake up when having to pass urine at night [8, 9]. However, studies indicate that much regarding the pathogenic mechanisms remain unknown [10]. It has been documented that nocturnal polyuria is common in children with NE [11] and can either be due to inadequate nocturnal arginine vasopressin (AVP) secretion during sleep or due to factors beyond renal water handling, such as osmotic diuresis and natriuresis [7, 11]. The circadian disturbance in plasma AVP and urine output seems, in a subset of enuresis patients, to occur only during some nights [12], the background of which is currently unknown.

In this hypothesis-free study, we investigated nocturnal urine samples from NE children with nocturnal polyuria and healthy children by using liquid chromatography-mass spectrometry (LC-MS)-based proteomics and metabolomics. Our aim was to identify metabolites and proteins that are different between a wet and a dry night to better understand the pathophysiology leading to a wet night and thereby the pathogenetic mechanisms behind this complex disorder.

## Material and methods

### Study design and patient inclusion

Ten boys (6–10 years of age) with primary monosymptomatic nocturnal enuresis (MNE) and nocturnal polyuria, who had never been treated for MNE, were recruited from the Center for Child Incontinence at Aarhus University Hospital (AUH), a tertiary referral center. The MNE diagnosis was based on a detailed clinical history, physical examination, and 2 weeks of home recordings. The inclusion criteria were a nocturnal urine production on wet nights above 130% of maximum voided volume (MVV) for age (MVV_age_ = 30 × [age in years + 1] ml [13]) at least one night per week [14] and a MVV above 65% of MVV_age_ [15] based on home recordings. The children needed to experience both wet and dry nights as comparisons were planned between these. Furthermore, ten healthy boys (9–14 years of age) without history of NE were included. Children with recurrent urinary tract infections, neurological or anatomical abnormalities of the urinary tract, or children who had undergone surgery in the urinary tract were excluded. No children included in the study suffered from nocturia, and no children were on any medication from 2 weeks before the urine collection.

For children with MNE, spot urine samples were collected before bedtime on both wet and dry nights. During sleep, urine was collected through a non-invasive collecting device (Uridome®). The first morning voided volume following both a wet and a dry night was also collected. The children continued this regimen until they had experienced both a dry and a wet night. The healthy children collected the first-morning void. Complete™, Mini Protease Inhibitor Cocktail (Roche Diagnostics, Mannheim, Germany) was added to the urine samples to minimize protein degradation. The children’s urine was tested for infection with a dipstick urinalysis. The urine was stored for 1 to 3 days in the freezer of the family, after which a fraction of the urine samples was stored at – 80 °C until analysis. Concentrations of sodium, potassium, creatinine, and calcium were measured in urine using routine procedures at the Dept. of Clinical Biochemistry, Aarhus University Hospital, Denmark. Urine osmolality was measured by the freezing point depression method (osmometer 3900, Advanced Instruments Inc., MA, USA).

### Preparation of samples for LC-MS analyses of metabolites

The urinary samples were diluted to obtain equal osmolality, and proteins in the urine (0.35 ml) were precipitated by addition of 1.4 ml – 20 °C methanol to obtain 80% methanol, followed by 30 s vortexing at 1200 RPM. The samples were stored overnight at – 20 °C whereafter the samples were centrifuged at 10,000 × *g* for 10 min, the supernatant was transferred to a new tube and evaporated to dryness, and stored at – 20 °C until LC-MS analysis of metabolites. Before LC-MS analyses, the dried samples were reconstituted in 0.2% formic acid and 3% methanol, vortexed for 15 min at 400 rpm, spun down, and subjected to ultrasonication for 5 s.

### Preparation of samples for LC-MS analyses of proteins

The urinary samples for proteomics were concentrated by a factor of ten by Centriprep® Ultrafiltration Centrifugal Filters (Merck Millipore, Cork, Ireland) with a molecular weight cut-off of 3000 Da, according to the manufacturer’s instructions. The protein concentration was determined by Bradford assay (BioRad), and amounts corresponding to 30 μg protein were precipitated overnight with six times sample volume acetone at − 20 °C, then centrifuged at 2600× g at 4 °C for 10 min, and finally air dried. The label-free proteomics analyses were essentially performed as previously described [16]. Briefly, protein pellets were dissolved in SDS-PAGE loading buffer and then subjected to SDS-PAGE followed by reduction and blocking of cysteines and in-gel trypsin digestion. The peptides extracted from the gel were subsequently purified with PepClean™ C18 Spin Columns according to the manufacturer’s instructions and then evaporated on a miVac Duo Concentrator (Genevac). Samples were stored at – 20 °C.

### LC-MS analyses of metabolites

The samples were analyzed by LC-MS on Vanquish LC and Q Exactive Plus Orbitrap MS, both from Thermo Scientific, essentially as previously described [17]. Autosampler was kept at 5 °C, and column temperature was 15 °C. A total of 10 μl of sample was injected, and the LC gradient constituted a 10-min gradient with 3–23% methanol, followed by washing and reequilibration. Both buffers A (LC-MS grade water) and B (LC-MS grade methanol) contained 0.2% formic acid. The analytical LC column was a 100-mm-long biphenyl column (Accucore, from Thermo Scientific) with inner diameter of 2.1 mm. The MS analyses were performed in both positive and negative MS mode at an electrospray voltage of 3500 V with scanning from 70 to 1050 *m/z*, and with the transfer tube heated to 300 °C. Stepwise fragmentation was performed with normalized collision energy levels of 20, 40, and 60. The MS was operated at high resolution (70,000) and accurate mass (< 5 parts per million) to assure high analytical selectivity.

Uric acid measurements were validated by parallel reaction monitoring (PRM) on a different mass spectrometer, Q Exactive HF-X (Thermo Scientific). The MS was operated in positive mode and the mass resolution was 60,000 in full scan mode and 15,000 for the fragmentation scans. Uric acid was quantified based on two transitions of parent *m/z* > fragment *m/z*, namely 169.036 > 141.04 and 169.036 > 152.01, defined based on analyses of an authentic standard (BioXtra, ≥ 99% HPLC grade, Merck) (Suppl. Fig. 1). Data from these analyses were treated in the Skyline software [18].

### LC-MS analyses of proteins

The peptide samples were analyzed by nano liquid chromatography (Easy-nLC 1200, Thermo Scientific)-tandem MS (Q-Exactive HF-X Hybrid Quadrupole Orbitrap, Thermo Scientific). Peptides were trapped by a pre-column (Acclaim PepMap 100 C18, pore size: 100 Å, particle diameter: 3 μm, inner diameter: 75 μm, and length: 2 cm, Thermo Scientific) and separated further with a reverse phase analytical column (PepMap RSLC C18, pore size: 100 Å, particle diameter: 2 μm, inner diameter 75 μm, and length 25 cm, Thermo Scientific) using an 80 min gradient from 5 to 90% AcN and 0.1% formic acid at a 270 nl/min flow rate. The mass spectrometer was operated in positive mode and higher collision dissociation (HCD), and normalized collision energy of 29 was used for peptide fragmentation. The full scan/MS1 resolution was 60,000, automatic gain control (AGC) target was 3 × 10^6^, maximum injection time was 80 ms, and scan range was from 352 to 1700 *m/z*. The fragmentation scan/MS2 resolution was 15,000. The MS was operated in data-dependent mode, and up to 12 of the most intense peaks were fragmented. Dynamic exclusion was set to 15 s. and unassigned and single-charge ions were excluded from fragmentation.

### Western blot analysis

Relative amounts of aquaporin 2 (AQP2) were validated by Western blot analyses, essentially as previously described [19]. The urine samples were separated on Criterion TGX Stain-free AnykD SDS-PAGE gels (Biorad). The primary antibody against Anti-Water Channel AQP2 was produced in rabbits (A7310 from Sigma-Aldrich), and the secondary antibody was polyclonal goat anti-rabbit antibody (DAKO, Glostrup, Denmark). Chemiluminescence and fluorescence detection of the membranes were performed to quantitate AQP2 and total protein stain (from the stain-free gels), respectively. ECL-kit (Thermo Scientific) was used according to the manufacturer’s recommendations, and the membranes were scanned on ImageQuant LAS 4000 (GE Healthcare). Quantification was performed in ImageQuant TL 7.0 (GE Healthcare).

### Data analysis

Compound Discoverer 3.3 (Thermo Scientific) was used for identification and quantification of small molecules in the urinary samples. The identification nodes were (1) predicted composition and the databases, (2) mzCloud (endogenous metabolites), and (3) ChemSpider (Human Metabolome Database, KEGG). The maximal allowed mass deviation between experimental MS data and database values was 5 parts per million. Proteins were identified and quantified using MaxQuant [20] (version 1.5.3.30) with its Andromeda algorithm against the human sequence database (*Homo sapiens* proteome with 20,129 reviewed sequences downloaded 16.12.2016 from Uniprot.org). Settings included: enzyme: trypsin, max missed cleavage sites: 2, precursor mass tolerance: 10 ppm, fragment mass tolerance: 0.02 Da, dynamic modification: oxidation, static modification: carbamidomethyl, false discovery rate (FDR): 0.01 at protein and peptide level. The “match between runs” were applied and iBAQ data were used for quantification. For each analysis, only compounds with quantitative values in 60% of the samples were included. For the included compounds, missing values were imputed by insertion of a value randomized between zero and the lowest one percentile of the dataset. Data were normalized so that each sample obtained the same total MS intensity.

Our main analysis was a comparison of metabolites and proteins in the urine from wet nights (urine from the uridome collected during sleep added to first morning voided volume) and from dry nights (first morning voided volume) in children with MNE. Metabolite and protein amounts were compared using a paired *t*-test (intra-individual comparison) on log2-transformed data (in Microsoft Excel 2016). Differences with *p*-values of < 0.05 and an average fold change (FC) < 0.67 or > 1.5 were considered statistically significant. *P*-values corrected for multiple testing were calculated by the Benjamini–Hochberg algorithm [21]. We also compared the urine samples collected before sleep preceding wet and dry nights in children with MNE. Finally, we compared the samples collected during wet and dry nights in children with MNE to samples collected from healthy children.

For functional grouping and annotation of differentially abundant proteins, analyses in the DAVID (The Database for Annotation, Visualization, and Integrated Discovery) [22, 23] and STRING database tool for functional protein association networks [24] were applied using default settings. For assessing tissue specificity and clustering of the differentially abundant proteins, the Functional Mapping and Annotation (FUMA) web application [25], GENE2FUNC, was applied using the GTEx v8 (54 tissue types) and otherwise default settings [26]. All quantified proteins were used as a background list.

Data in Tables 1 and 2 were normally distributed between groups and handled as paired data. *P*-values of < 0.05 were considered significant. Analyses were performed using Stata IC, version 15.0.

**Table 1:** The basic demographic data of the study subjects, mean ± standard deviation or percentages

	Children with enuresis	Healthy children
Age (years)	7.6 ± 1.3	11.7 ± 1.7
Height (cm)	133 ± 0.12	155 ± 0.14
Weight (kg)	31.2 ± 9.4	43.3 ± 11.7
BMI (kg/m^2^)	17.5 ± 2.1	17.7 ± 2.2
Enuresis frequency/week (*n*)	5.3 ± 1.5	
Mean nocturnal urine production on wet nights based on home-recordings (ml)*	360 ± 65	
Maximum voided volume based on home-recordings (*% of expected MVV*_age_**)	100 ± 38%	
Nocturnal urine output collected during the wet night (*% of expected MVV*_age_**)	156 ± 50%	
Nocturnal urine output collected during the dry night (*% of expected MVV*_age_**)	75 ± 23%	82 ± 27%

*Mean nocturnal urine production on wet nights excluding nights with nocturia based on 14 days measurements. *n*, number; *MVV*_*age*_, maximum voided volume for age [13]

**Table 2:** Biochemical measurements of urine samples, mean ± standard deviation, *P < 0.05

	Wet nights, enuresis	Dry nights, enuresis	*P-value* *(Wet nights vs. dry nights, enuresis)*	Healthy children	*P-value* *(Wet nights vs. healthy children)*	*P-value* *(Dry nights vs. healthy children)*
U-sodium (mmol/kg BW)	1.42 ± 0.88	0.73 ± 0.55	*0.19*	0.77 ± 0.43	*0.070*	*0.86*
U-potassium (mmol/kg BW)	0.43 ± 0.25	0.20 ± 0.11	0.038*	0.24 ± 0.14	*0.058*	*0.53*
U-calcium (mmol/kg BW)	0.020 ± 0.013	0.018 ± 0.014	*0.36*	0.012 ± 0.012	*0.18*	*0.36*
U-creatinine (mmol/kg BW)	0.062 ± 0.030	0.055 ± 0.015	*0.44*	0.060 ± 0.024	*0.88*	*0.58*
U-osmolality (mosmol/kg)	588 ± 197	773 ± 223	0.025*	683 ± 239	*0.38*	*0.40*
U-excretion osmoles (mosmol/kg BW)	7.99 ± 3.22	5.16 ± 2.39	*0.074*	4.73 ± 2.16	0.026*	*0.69*

The local Danish Research Ethics Committee and the Danish Data Protection Agency approved the study, and the study was registered at ClinicalTrials.gov (NCT04049019). The children and their parents were informed orally and in writing about the project, and both oral and written consent were obtained from custody holders of the child before inclusion.

## Results

### Patient characteristics

Ten boys with MNE (7.6 ± 1.3 years of age) and ten boys without NE (11.7 ± 1.7 years of age) succeeded in collecting urine as per protocol (Table 1). After the end of the study, nine of the ten children with MNE were treated with desmopressin (DDAVP) 240 μg in our outpatient clinics. Of these children, one child fully responded to DDAVP, six children experienced partial response (according to ICCS criteria with more than 50% reduction in wet nights [15]), and two children experienced no response. The last child responded to alarm treatment as first-line treatment.

### Nocturnal urine output

The mean nocturnal urine production during wet nights was 156 ± 50% of expected MVV_age_, compared with 75 ± 23% of expected MVV_age_ during dry nights for children with MNE, and 82 ± 27% of expected MVV_age_ in the healthy children (Table 1). The creatinine excretion was similar during wet and dry nights and comparable to the levels of healthy children (Table 2). For children with MNE, the urine osmolality was significantly lower during wet nights compared to dry nights (588 ± 197 vs. 773 ± 223 mosmol/kg, *P* = 0.025), whereas urine osmolality during dry nights was comparable to values from healthy children (Table 2). Sodium excretion (1.42 ± 0.88 vs. 0.73 ± 0.55 mmol/kg BW, *P =* 0.19) and potassium excretion (0.43 ± 0.25 vs. 0.20 ± 0.11 mmol/kg BW, *P* = 0.038) were markedly higher, though only statistically significant for potassium, on wet nights compared to dry nights, whereas the dry nights were comparable to controls (Table 2). We found the sodium/potassium ratio to be lower on wet nights compared to dry nights; however, the difference was small and not statistically significant (*P* = 0.59). The calcium excretion was not significantly different between wet and dry nights (0.018 ± 0.014 vs. 0.020 ± 0.013 mmol/kg BW, NS) (Table 2).

### Metabolomics

**Fig. 1:**
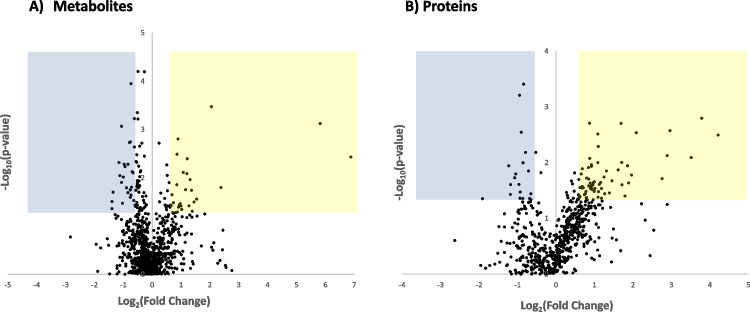
Volcano plot of **A** metabolomics data and **B** proteomics data. Negative log_10_ (*p-*value) plotted versus log_2_ (fold change) for all quantified metabolites and proteins. The blue and yellow quadrants indicate downregulated and upregulated areas, respectively, based on both *p*-value (*P* < 0.05) and fold change criteria (ratio < 0.67 or > 1.5)

Creatinine was quantified by untargeted LC-MS analyses of metabolites and deviated less than 6% between samples from wet and dry nights in children with MNE (and in the same direction as biochemical measurements), which indicates that a direct comparison of the amounts of metabolites in samples from wet and dry nights after normalizing of data is reasonable. MS identified and quantified 642 metabolites in positive mode and 262 metabolites in negative mode (Fig. 1A, Suppl. Table 1). Among these, 59 metabolites were present in significantly different amounts between the MNE wet and dry nights with FC < 0.67 or > 1.5 and *P* < 0.05 (Suppl. Table 2). Levels of five metabolites were significantly different when comparing wet and dry nights and correcting for multiple testing (Suppl. Table 2).

**Fig. 2:**
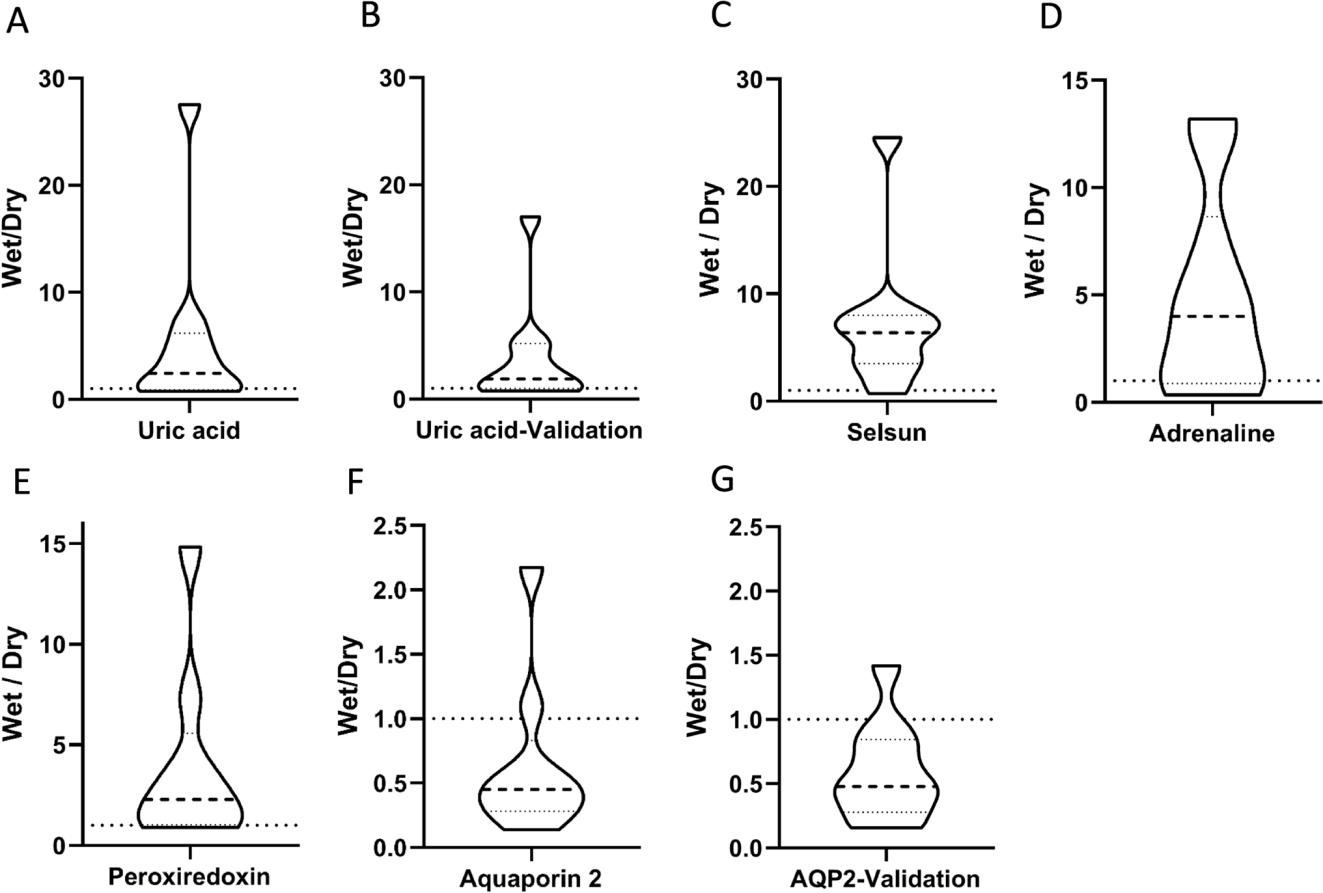
Violin plots of the fold change (ratio) of quantitative mass spectrometry intensities of compounds when comparing wet and dry nights (nocturnal urine production) for children with enuresis. Broken lines indicate median and dotted lines indicate quartiles. The value 1.0 on *y*-axis, corresponding to unaltered level in wet versus dry nights, is indicated by a horizontal dotted line. Levels of uric acid (**A**, **B**), selsun (**C**), adrenalin (**D**), and peroxiredoxin (**E**) were increased, and levels of aquaporin 2 (AQP2) were decreased (**F**, **G**). Uric acid concentrations were validated by independent mass spectrometry analyses applying parallel reaction monitoring (PRM) (**B**). Levels of aquaporin 2 (AQP2) were validated by western blot analyses (**G**)

More than 20 metabolites, which were all significantly increased during wet nights compared to dry nights, were identified as relevant in relation to oxidative stress in the body, among others uric acid and selenium (identified as selsun and selenium sulfide) (Fig. 2), as well as lanthionine ketimine, methylsulfonylmethane, s-adenosylmethionine, vitamin C, and vitamin B5 (Suppl. Table 2). Levels of uric acid were also significantly different between wet and dry nights when correcting for multiple testing (Suppl. Table 2). Uric acid concentrations were validated by independent, targeted PRM LC-MS/MS analyses, designed based on data from a commercial authentic standard sample (Fig. 2, Suppl. Fig. 1). The average FC of uric acid in wet versus dry nights was 2.91 (*P* = 0.028) and 2.23 (*P* = 0.040) in the omics and validation PRM analysis, respectively. Furthermore, the level of adrenalin was found to be 2.4 times higher on wet nights compared to dry nights, *P* = 0.038 (Fig. 2).

Among the metabolites detected in urine samples collected before sleep in children with MNE, 28 were significantly altered when comparing samples preceding wet and dry nights using the same criteria as above (Suppl. Table 3). The subset of the metabolites that were differentially altered during wet versus dry nights (*n* = 59) had FCs that correlated with the FCs of the same set of metabolites observed in the urine samples collected before sleep (*R*^*2*^ = 0.37) (Suppl. Fig. 2A). Based on the list of metabolites significantly different between wet and dry nights (positive mode MS), metabolites from the samples collected before sleep in children with MNE (wet/dry FC) and the metabolites from the samples from the nocturnal urine production (wet/dry FC) were hierarchically clustered (Suppl. Fig. 3). This revealed that the evening samples (wet/dry FC) clustered separately and were less homogeneous than the samples from the nocturnal urine production (wet/dry FC).

**Fig. 3:**
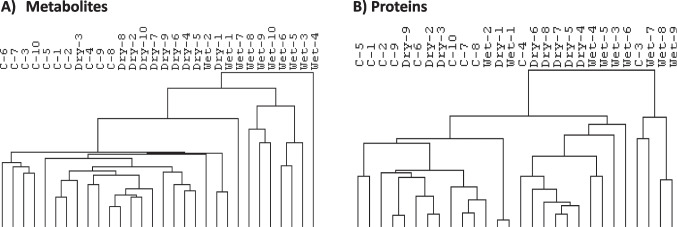
Cluster tree from hierarchical clustering analysis of **A** metabolites and **B** proteins significantly different between wet and dry nights in enuresis samples based on quantitative mass spectrometry intensities. Control (C) samples accumulate mainly in the left part of the cluster tree, and samples from wet nights (wet) from children with enuresis cluster to the right. C = nighttime urine production of healthy children; dry = nighttime urine production from dry nights (children with enuresis); wet = nighttime urine production from wet nights (children with enuresis)

We further identified 68 metabolites with levels that were significantly different between wet nights in children with MNE and nights of healthy children (FC < 0.67 or > 1.5, and *P* < 0.05), here among compounds relevant for protection against oxidative stress, including lanthionine ketimine, s-adenosylmethionine, and glutarylcarnitine (Suppl. Table 4). Based on the list of metabolites significantly different between wet and dry nights (*n* = 59), we performed hierarchical clustering of metabolites from samples from wet nights (MNE), dry nights (MNE), and the samples from healthy children based on the MS intensities of metabolites (Fig. 3A). This revealed clustering of the samples from the dry nights in children with MNE next to the samples from the healthy children.

### Proteomics

The untargeted LC-MS analyses of urine proteins were based on samples from nine children with MNE, since the MS signal was insufficient for one child.

MS identified and quantified 620 proteins (Fig. 1B, Suppl. Table 5). These proteins were enriched for proteins with the GOterm “extracellular matrix,” for proteins being expressed in the medullary kidney, and for proteins contained in the glomerular matrisome (*n* = 115) [27]. Of the 620 proteins quantified, 84 were present at levels significantly different between wet and dry nights in children with MNE with FC < 0.67 or > 1.5 and *P* < 0.05 (Suppl. Table 6). The DAVID Bioinformatics Resources [22, 23] was used to analyze for functional overrepresentations. Three clusters were significant in the analyses: (1) proteins with immunoglobulin domain, (2) calcium ion-binding proteins, and (3) glycoproteins (Suppl. Table 9). DAVID analyses did not point at enrichment for any specific cellular compartments (GOTERM_CC_DIRECT). GEN2FUNC [25, 26] analyses of the same set of proteins using the GTEx v8 expression data did not identify any tissue expression specificity of the proteins that were differentially abundant between wet and dry nights; however, clusters were apparent among proteins with high expression in blood, liver, skin, brain, and kidneys (Suppl. Fig. 4). Differentially abundant proteins were neither enriched for proteins in the glomerular matrisome (*P* = 0.23, Fisher’s exact test) [27].

**Fig. 4:**
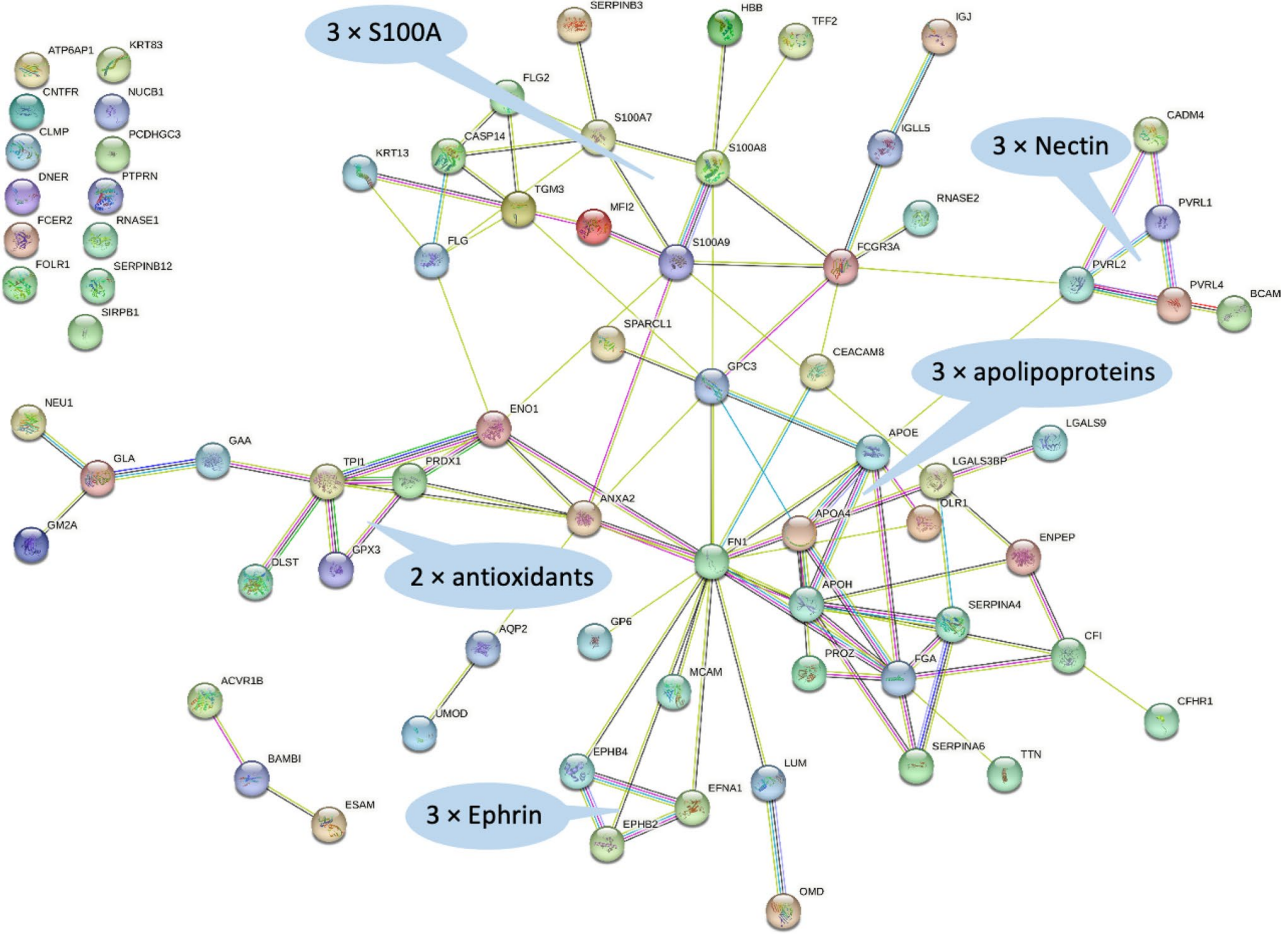
STRING database tool [24] for functional pathway analysis (STRING: functional protein association networks (string-db.org)) of proteins significantly different (fold change < 0.67 or > 1.5, *P* < 0.05) between wet and dry nights in children with enuresis. Thirteen proteins, upper left corner, did not have connectivity using default STRING settings. Groups of proteins are highlighted (blue bubbles)

Based on the group of proteins significantly different between wet and dry nights in children with MNE (*n* = 84), STRING analyses [24] (Fig. 4) confirmed the results from the metabolomics study by identifying functional similarities between proteins important for oxidative stress in the body, including peroxiredoxin-1 (FC = 1.95, *P =* 0.02), which plays a role as an antioxidant protective enzyme (Fig. 2). The DAVID Bioinformatics Resources and STRING analyses pointed at calcium-binding proteins (Fig. 4, Suppl. Table 9). This group of proteins is rather heterogeneous, including among others nucleobindin-1 (FC = 1.69, *P =* 0.036), a major calcium-binding protein of the Golgi apparatus, three S100 proteins (Fig. 4), and upregulated amounts of uromodulin (the latter being upregulated with FC = 1.53, *P =* 0.028), which among many roles is described as an inhibitor of calcium crystallization in renal fluids [28]. STRING analyses identified another group of proteins with relevance for cell adhesion, including three nectins (PVRL1, 2, and 4), basal cell adhesion molecule (BCAM), and cell adhesion molecule 4 (CADM4), all found in increased levels (Fig. 4, Suppl. Table 6). Furthermore, three proteins of the ephrin signaling pathway were grouped by STRING analysis that are involved in cell migration and tissue development through its regulation of cell-cell adhesion [29] (Fig. 4). STRING also identified a group of proteins with relevance for lipid metabolism, including apolipoprotein A and E (FC = 3.68*, P =* 0.023 and FC = 2.29, *P =* 0.049) and lipoprotein receptor 1 (FC = 2.63, *P =* 0.025) (Fig. 4).

Of further note, three proteins described as relevant for blood pressure regulation were identified when comparing wet and dry nights in children with MNE: kallistatin (FC = 2.14, *P =* 0.005), glutamyl aminopeptidase (FC = 1.80, *P =* 0.02), and fibronectin, the last, however, downregulated (FC = 0.64, *P =* 0.036) (Suppl. Table 6). We found the levels of AQP2 to be 0.44 times lower on wet nights compared to dry nights (*P =* 0.025) (Fig. 2). During the wet nights, we found a correlation between AQP2 levels and urine osmolality (*R*^*2*^ = 0.50). Based on the Western blot intensity data, levels of AQP2 (average FC = 0.59, *P* = 0.02) were reduced when comparing wet and dry nights (Fig. 2, Suppl. Fig. 5). Levels of corticosteroid-binding globulin, which can bind serum aldosterone, were increased during wet nights compared to dry nights (FC = 1.89, *P =* 0.01) (Suppl. Table 6).

In total, the levels of 21 proteins were significantly different in samples collected before sleep preceding wet and dry nights in children with MNE using the same criteria as above (Suppl. Table 7). The subset of the proteins that were differentially altered during wet versus dry nights (*n* = 84) did not have FCs that correlated with the FCs of the same set of proteins observed in the urine samples collected before sleep (*R*^2^ = 0.06) (Suppl. Fig. 2B). Based on the list of proteins significantly different between wet and dry nights (*n* = 84), the proteins from the samples collected before sleep in children with MNE (wet/dry FC) and the proteins from samples from the nocturnal urine production (wet/dry FC) were hierarchically clustered (Suppl. Fig. 6). In this analysis, the evening samples (wet/dry FC) clustered separately and were less homogeneous than the samples from the nocturnal urine production (wet/dry FC).

Supplementary Table 8 presents the list of six proteins with significantly different levels between wet nights in children with MNE and nights in healthy children (FC < 0.67 or > 1.5, and *P* < 0.05). Based on the list of proteins significantly different between wet and dry nights (*n* = 84), we performed hierarchical clustering of proteins from samples from wet nights (MNE), dry nights (MNE), and samples from healthy children based on the MS intensities of proteins (Fig. 3B). This revealed clustering of the samples from the dry nights in children with MNE next to the samples from the healthy children.

## Discussion

In this hypothesis-free study, we performed MS-based metabolomics and proteomics on urine samples from well-characterized children with MNE and nocturnal polyuria and healthy children. Among MS-identified metabolites and proteins, attention should be pointed to compounds relevant for oxidative stress and adrenalin, as levels were found significantly increased during wet nights compared to dry nights. Proteins with relevance for blood pressure regulation were also different between wet and dry nights. The level of AQP2, which by AVP mediates reabsorption of water in the collecting duct in the kidneys [30], was reduced on wet nights (also validated), whereas levels of AQP2 during dry nights were similar to the levels in samples from healthy children. The FCs of metabolites significantly different between wet and dry nights positively correlated to the FCs in the same metabolites identified in urine samples obtained during the evening preceding wet versus dry nights. Bioinformatics analyses did not point at enrichment for any specific cellular or tissue origin of the proteins that differed between wet and dry nights in children with MNE; however, a subset of the proteins belonged to groups with high expression in blood, liver, skin, brain, and kidneys.

Oxidative stress is defined as an excessive production of reactive oxygen species (ROS) that cannot be countered by the action of antioxidants [31]. We identified increased amounts of metabolites and proteins related to oxidative stress during wet nights compared to dry nights in children with MNE, here among well-described antioxidants such as vitamin C, selenium, and peroxiredoxin 1. The increased levels of antioxidants could indicate compensatory mechanisms for stress induction. Oxidative stress and NE have not been linked before, but the connection between oxidative stress and nocturia in adults has been a matter of debate for years [32]. Matsumoto et al. showed in a cross-sectional study of 1113 adults that the amount of advanced glycation end products (AGEs) measured by skin autofluorescence, surrogate markers of oxidative stress, were significantly associated with the frequency of nocturia [33]. Konishi et al. found oxidative stress to be significantly associated with worsening sleep disorders, although not with worsening nocturia frequency [34]. It is known that NE and nocturia share common pathophysiology pathways [35]. Research highlights the role of circadian dysfunction in urine production together with sleep disturbances such as fragmentation and periodic limb movements in NE children [9], and sleep disturbances and circadian dysfunction are described as causes of oxidative stress [36, 37]. Together with markers for oxidative stress, we found a 2.4-fold increase in adrenalin levels during wet nights and the presence of proteins relevant to blood pressure regulation. Kruse et al. provided evidence of an increased nocturnal mean arterial pressure in NE children with polyuria and described a positive correlation between average nocturnal mean arterial pressure and nocturnal urine volume [38], findings that have been confirmed in other studies [39]. Further in line with this, we found increased levels of uromodulin (also in validation experiments) on wet nights compared to dry nights, which seems relevant since uromodulin is described to be important in sodium handling, blood pressure regulation, and modulation of oxidative stress in the body [40]. One could hypothesize that part of the underlying mechanism for increased sympathetic activity and stress during wet nights is sleep disturbances. Or the other way around, as Dhondt et al. suggested—the increased sympathetic activity and increased activity in our dopaminergic system influence cortical arousals and sleep in children with NE [9]. Gaby et al. performed standard polysomnography and recordings of body movements in children with NE. They found that tachycardia and autonomous arousal often are present before an enuresis event [41]. The autonomic nervous system also plays an essential role in kidney function, and it is described how renal innervation influences the amount and constituents of urine [42].

Another important factor in water and blood pressure regulation is the renin–angiotensin–aldosterone system. Rittig et al. reported lack of circadian rhythm in serum aldosterone and plasma angiotensin II in children with NE and nocturnal polyuria [43]. We found an increase in corticosteroid-binding globulin during wet nights, which could reflect lower aldosterone levels. Since corticosteroid-binding globulin also binds other steroid hormones, such as cortisol, low aldosterone level is solely a hypothesis that needs further elucidation. Furthermore, the sodium/potassium ratio was not statistically different on wet nights compared to dry nights. Harrison et al. reported how hypertensive stimuli, such as salt intake and angiotensin II, seem to promote the production of ROS in the brain, the kidney, and the vasculature, which together with sympathetic activity contributes to an increase in arterial blood pressure [44]. Oxidative stress, sleep disturbances, and increased sympathetic activity together with the renin–angiotensin–aldosterone system might be important in NE pathophysiology and nocturnal polyuria; however, it is unknown what is cause and what is the consequence. If studies confirm increased oxidative stress during wet nights, research should clarify if oxidative stress, sleep disturbances, and increased blood pressure during the night have any long-term consequences in children with NE.

It is well described that at least some children with nocturnal polyuria have increased sodium excretion during wet nights, and that nocturnal polyuria is not only explained by AVP alterations [45, 46], which is in line with our findings of increased but not significant sodium excretion. This is supported by the observation that only one child experienced full response to DDAVP treatment after urine collection. Unfortunately, we do not have information about nutritional and fluid intake, which might affect osmotic excretion and nocturnal urine production [47]. Standardization of nutritional and fluid intake should be practiced to fully evaluate renal osmotic handling in children with nocturnal polyuria.

The protein data revealed a rather large group of calcium-binding proteins, all in increased levels during wet nights compared to dry nights. Though related, the proteins in this group appeared rather heterogeneous with respect to their function. Studies have pointed at hypercalciuria as an important factor in the pathogenesis of MNE [48, 49], however, this could not be confirmed in 46 Danish children with desmopressin-resistant MNE [50]. In our analysis, the urinary calcium excretion did not differ significantly between wet and dry nights for children with MNE. None of the identified calcium-binding proteins, though, seemed to have a direct role in calcium excretion; thus, more research is needed to clarify the role of calcium and calcium-binding proteins in relation to polyuria in children with MNE.

We identified several proteins with relevance to cell adhesion. One could speculate how cell adhesion in the kidneys could influence water and salt handling and how expansion of the bladder and increased urine production may affect levels of cell adhesion molecules. Finally, we found an increase in proteins relevant to lipid metabolism and immune system (immunoglobulins); however, the relevance of these findings in NE pathophysiology is not obvious.

We found the FCs of metabolites significantly different between wet and dry nights to be positively correlated to the FCs in the same metabolites identified in urine samples obtained during the evening preceding wet versus dry nights. In relation to these findings, we evaluated the spot urine samples collected at bedtime in children with MNE and found a tendency toward a lower urine osmolality before wet nights than before dry nights (730 ± 232 vs. 876 ± 141 mosmol/kg, NS), and higher urinary sodium and potassium excretions before wet nights than before dry nights (sodium: 1.13 ± 0.28 vs. 0.77 ± 0.17 mmol/mmol creatinine/kg BW, NS, potassium: 0.24±0.049 vs. 0.15 ± 0.044 mmol/mmol creatinine/kg BW, NS). Despite less homogeneous evening samples, our results could indicate that some of the pathophysiological mechanisms causing a wet night are present already during the evening hours. This is supported by results recently published by Karamaria et al., which concluded that the overall 24-h diuresis and osmotic excretion play a role in NE [51]. When evaluating urine composition, dry nights seem to be comparable to nights of children without a history of NE.

One important limitation of this study is the limited number of participants. Thus, our results should be confirmed in a larger study population of boys and girls. Intra-individual comparison was not possible since the children collected urine during one wet and one dry night. In pooled urine samples, Sun et al. found 24 proteins to be present in relatively constant amounts between days and between individuals [52]. Among these, four were identified to be present in significantly different amounts between wet and dry nights in our study, including uromodulin and apolipoprotein H. Another limitation is the age difference of on average 4 years between children with MNE and healthy children. We therefore adjusted urine measurement according to body weight. The urine samples from the wet nights in children with MNE were stored in the collecting devices during the night, while the urine samples from the dry nights (MNE) and from the healthy children were stored in the bladder, which could influence the constituents of the urine. We did not have information about the specific period (hours) in which the nighttime urine samples were collected. The families were asked to register urine volumes, possibly introducing measurement bias. The MS analysis has a selection bias toward detection of the abundant molecules, so although data on hundreds of compounds are reported, not all metabolites and proteins in the urine were mapped out. Furthermore, the risk of false positive data is a known limitation in large-scale MS studies, which we tried to minimize by using both FC and *p*-value criteria. Furthermore, some compounds were validated by different methodologies.

## Conclusion

We performed MS-based metabolomics and proteomics in children with MNE and nocturnal polyuria and validated some compounds by different methodologies. Our results indicate that oxidative stress levels might be increased during wet nights, and we speculate how this could be related to disturbances in circadian rhythm and sleep in NE children. We point to the importance of the sympathetic nervous system based on the findings of higher adrenalin levels on wet nights. We also found a reduced level of AQP2 and an increase in corticosteroid-binding globulin (which binds aldosterone), combined with increased sodium and potassium excretion during wet nights compared to dry nights. Our results support that the mechanisms causing a wet night during sleep in NE children are complex and indicate that both free water and solute handling are important.

## Supplementary information

Graphical Abstract (PPTX 411 KB)

ESM 1 (PDF 609 KB)

ESM 2 (XLSX 484 KB)
