# Supplementary material for: Differences in the urinary metabolome and proteome between wet and dry nights in children with monosymptomatic nocturnal enuresis and nocturnal polyuria
Source: Pediatr Nephrol. 2023 May 4;38(10):3347–58. doi: 10.1007/s00467-023-05963-5 (PMC10465629; doi:10.1007/s00467-023-05963-5)
Supplement: Supplementary file 1 — Graphical Abstract (PPTX 411 KB) [file 467_2023_5963_MOESM1_ESM.pptx]

## Slide 1
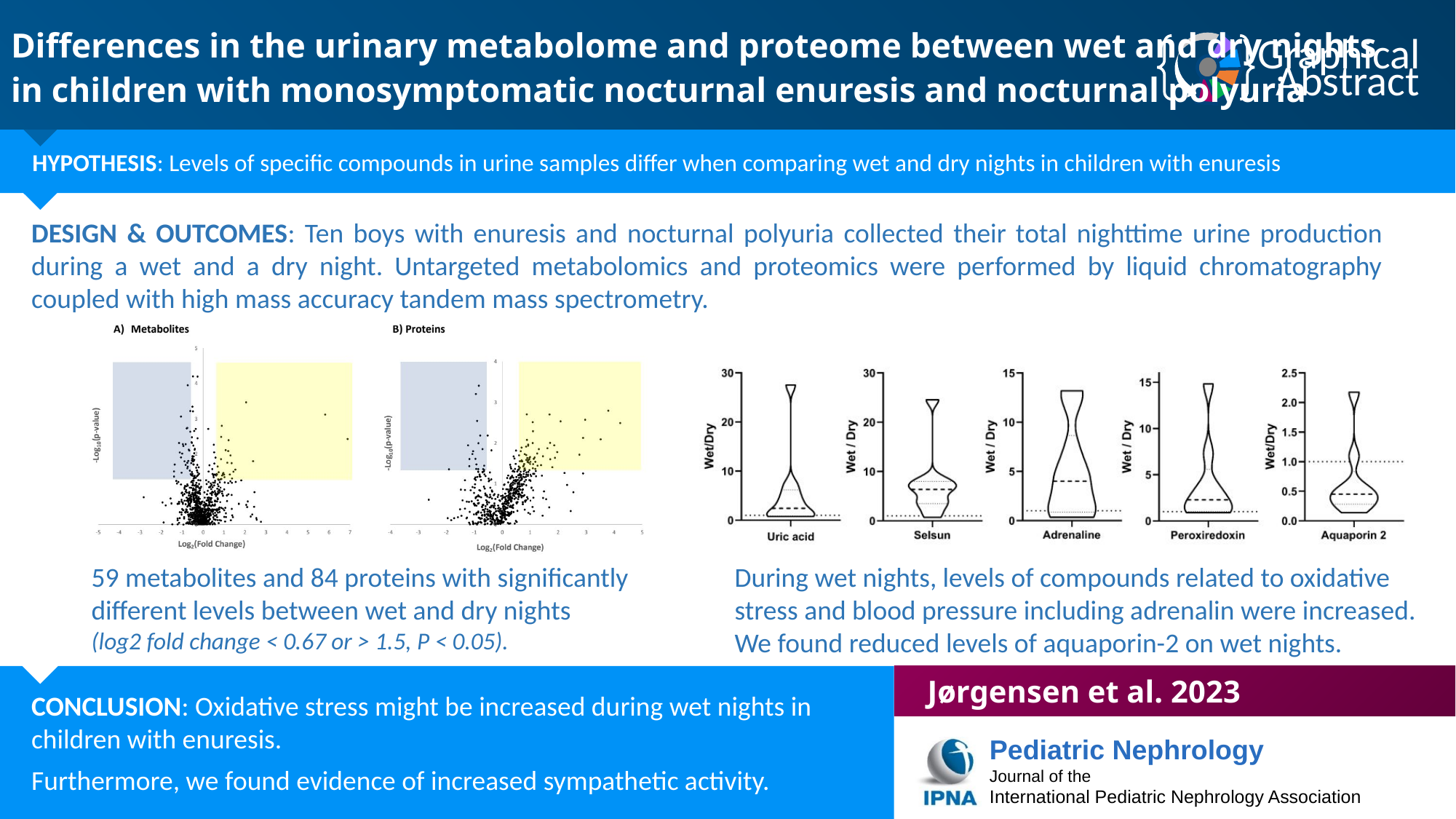

Differences in the urinary metabolome and proteome between wet and dry nights
in children with monosymptomatic nocturnal enuresis and nocturnal polyuria
HYPOTHESIS: Levels of specific compounds in urine samples differ when comparing wet and dry nights in children with enuresis
DESIGN & OUTCOMES: Ten boys with enuresis and nocturnal polyuria collected their total nighttime urine production during a wet and a dry night. Untargeted metabolomics and proteomics were performed by liquid chromatography coupled with high mass accuracy tandem mass spectrometry.
During wet nights, levels of compounds related to oxidative stress and blood pressure including adrenalin were increased. We found reduced levels of aquaporin-2 on wet nights.
59 metabolites and 84 proteins with significantly
different levels between wet and dry nights
(log2 fold change < 0.67 or > 1.5, P < 0.05).
Jørgensen et al. 2023
CONCLUSION: Oxidative stress might be increased during wet nights in children with enuresis.
Furthermore, we found evidence of increased sympathetic activity.
