# Supplementary material for: Differences in the urinary metabolome and proteome between wet and dry nights in children with monosymptomatic nocturnal enuresis and nocturnal polyuria
Source: Pediatr Nephrol. 2023 May 4;38(10):3347–58. doi: 10.1007/s00467-023-05963-5 (PMC10465629; doi:10.1007/s00467-023-05963-5)
Supplement: Supplementary file 2 — ESM 1 (PDF 609 KB) [file 467_2023_5963_MOESM2_ESM.pdf]

Supplementary Figures

Differences in the urinary metabolome and proteome between wet and dry nights in children with monosymptomatic nocturnal enuresis and nocturnal polyuria

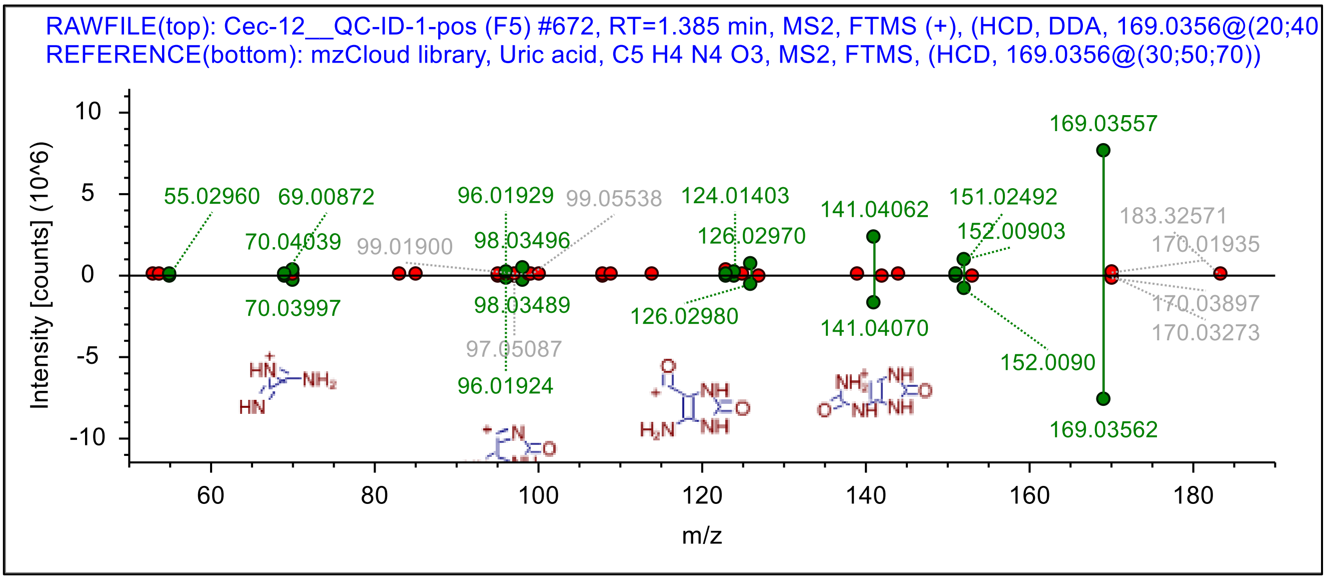

Supplementary Figure 1. Mass spectrometric fragmentation spectrum of uric acid. The fragments detected in patient samples (top) matched the mirrored library fragments (below) of authentic standard. The peak with m/z value of 169.036 corresponds to unfragmented parent ion. The two most dominating fragments, 141.04 and 152.01, were used as signature fragments in the quantitation experiments using parallel reaction monitoring (PRM) liquid chromatography mass spectrometry.

A)

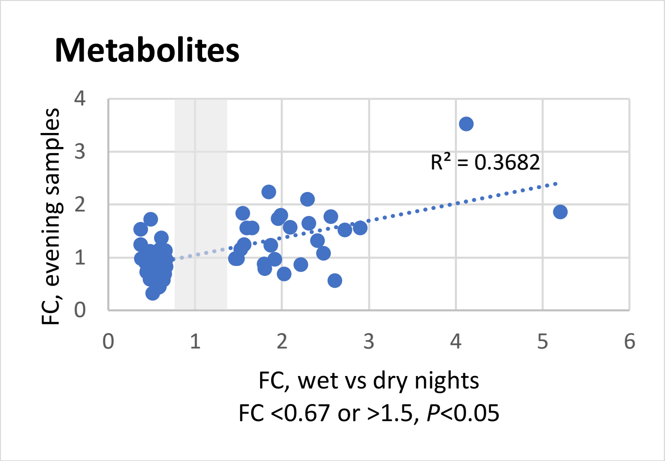

B)

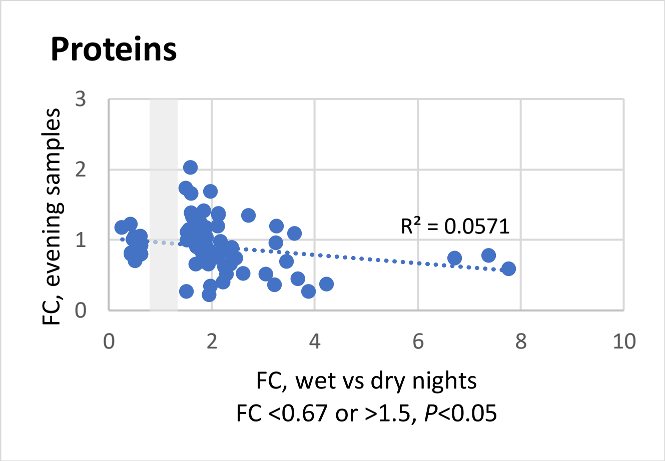

Supplementary Figure 2. Correlation plot of compounds from samples obtained during the evening preceding wet/dry nights plotted against samples from wet/dry nights. Compounds significantly altered during wet versus dry nights are plotted (fold change (FC)>1.5 or <0.67, P<0.05). Plot A (metabolites) indicates a correlation (R^2=0.37).

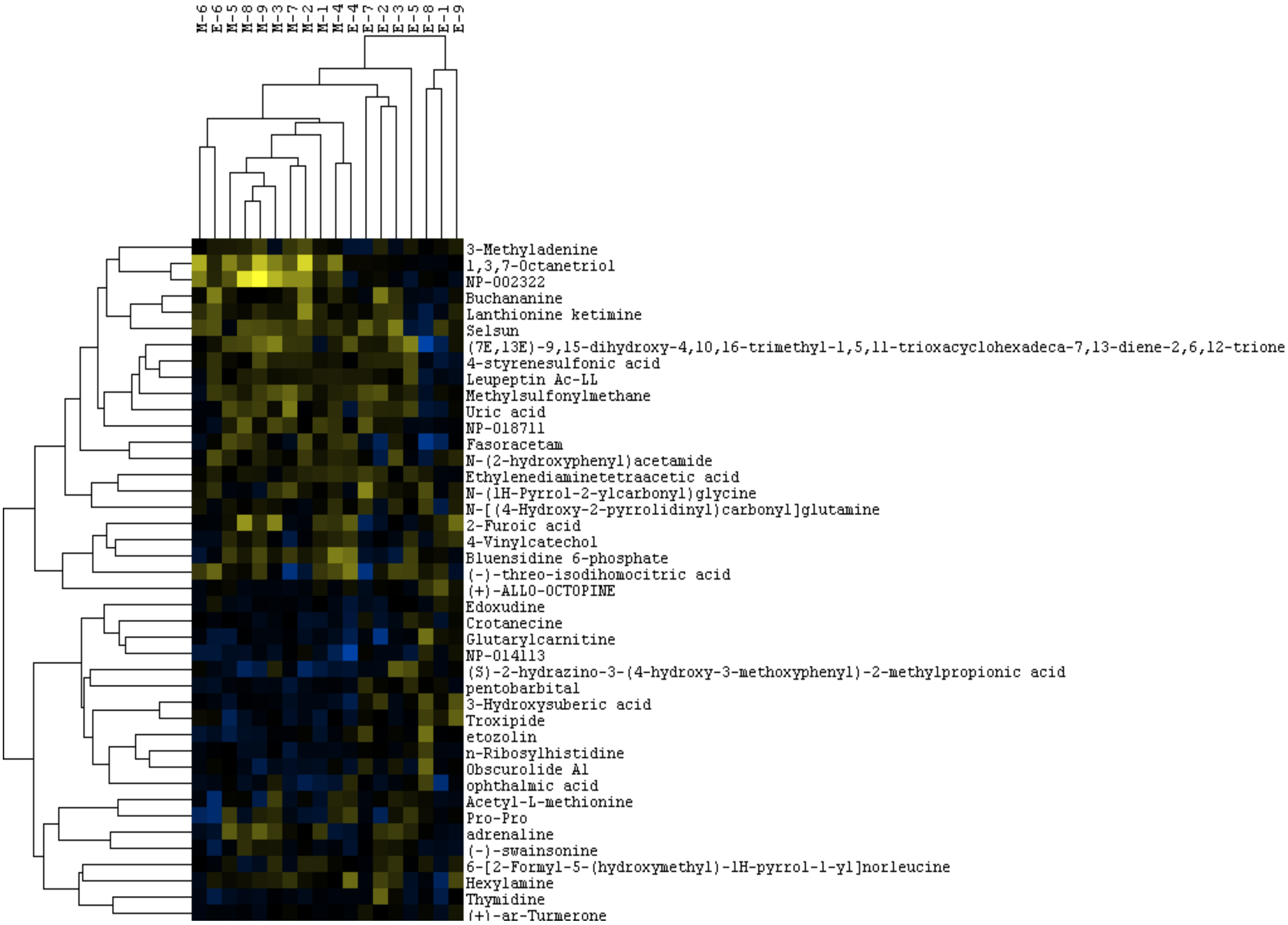

Supplementary Figure 3. Hierarchical cluster of metabolite ratios (positive mode mass spectrometry) with full cluster. Depicted are up- (yellow) and downregulated (blue) metabolites when clustering metabolites significant different between wet and dry nights in children with enuresis (fold change (FC)>1.5 or <0.67, *P*<0.05). The evening values cluster to the right and are less homogeneous.

M (morning) = Wet / Dry ratio (FC) in nocturnal urine production from children with enuresis.

E (evening) = Wet / Dry ratio (FC) in urine samples collected before sleep from children with enuresis.

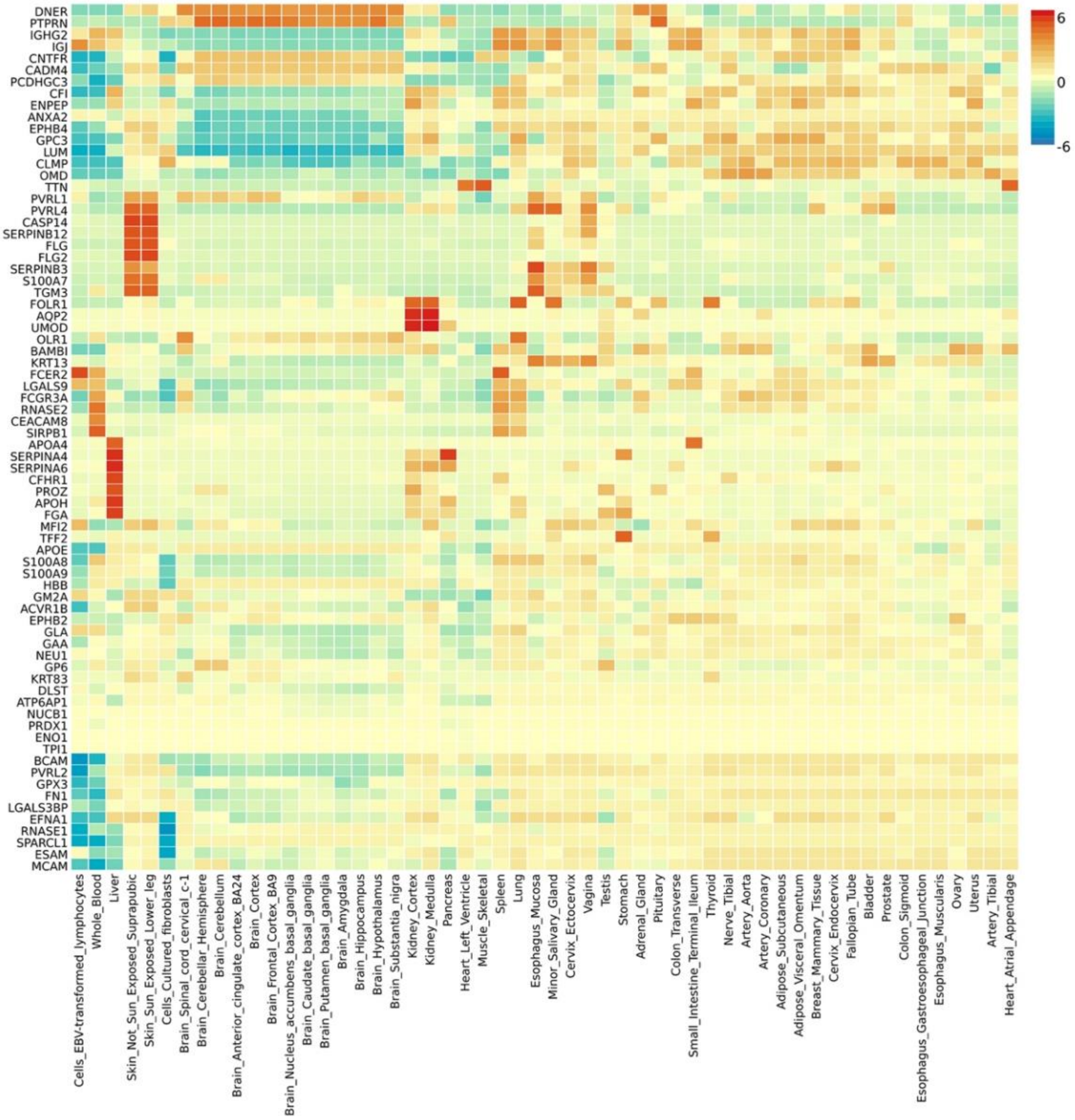

Supplementary Figure 4. Human gene expression heatmap of the genes corresponding to the proteins identified to be present in different amounts between wet and dry nights in enuresis children. The Functional Mapping and Annotation of Genome-Wide Association Studies (FUMA GWAS) GENE2FUNC [21] online tool was used to create a heatmap of the gene expression using log2 transformed expression values from the Genotype-Tissue Expression (GTEx) v8 [22] from 54 general tissue types. Red cells depict higher expression compared to cells filled in blue and yellow represents expression that is not significantly different from other genes. The values depicted are the average of the zero mean normalized expression per tissue type and the heatmap was clustered both at the gene and tissue type level.

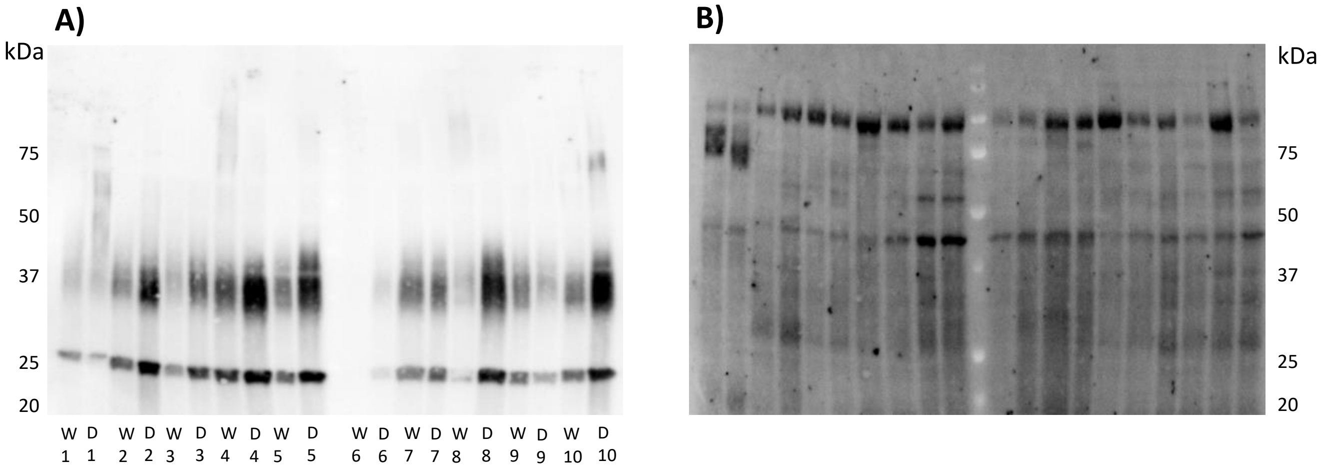

Supplementary Figure 5. Figure A illustrates Western blot detection of aquaporin 2 (AQP2) in urinary samples from Wet (W) vs Dry (D) nights in children with enuresis. AQP2 is detected as glycosylated (30-45 kDa) and non-glycosylated (26 kDa), and band intensity of those two regions were integrated separately and then summed. Figure B depicts the total protein stain of the membrane. AQP2 signal (A) was, for each sample, divided by the total protein intensity of the corresponding lane to normalize for possible variation in loading amount.

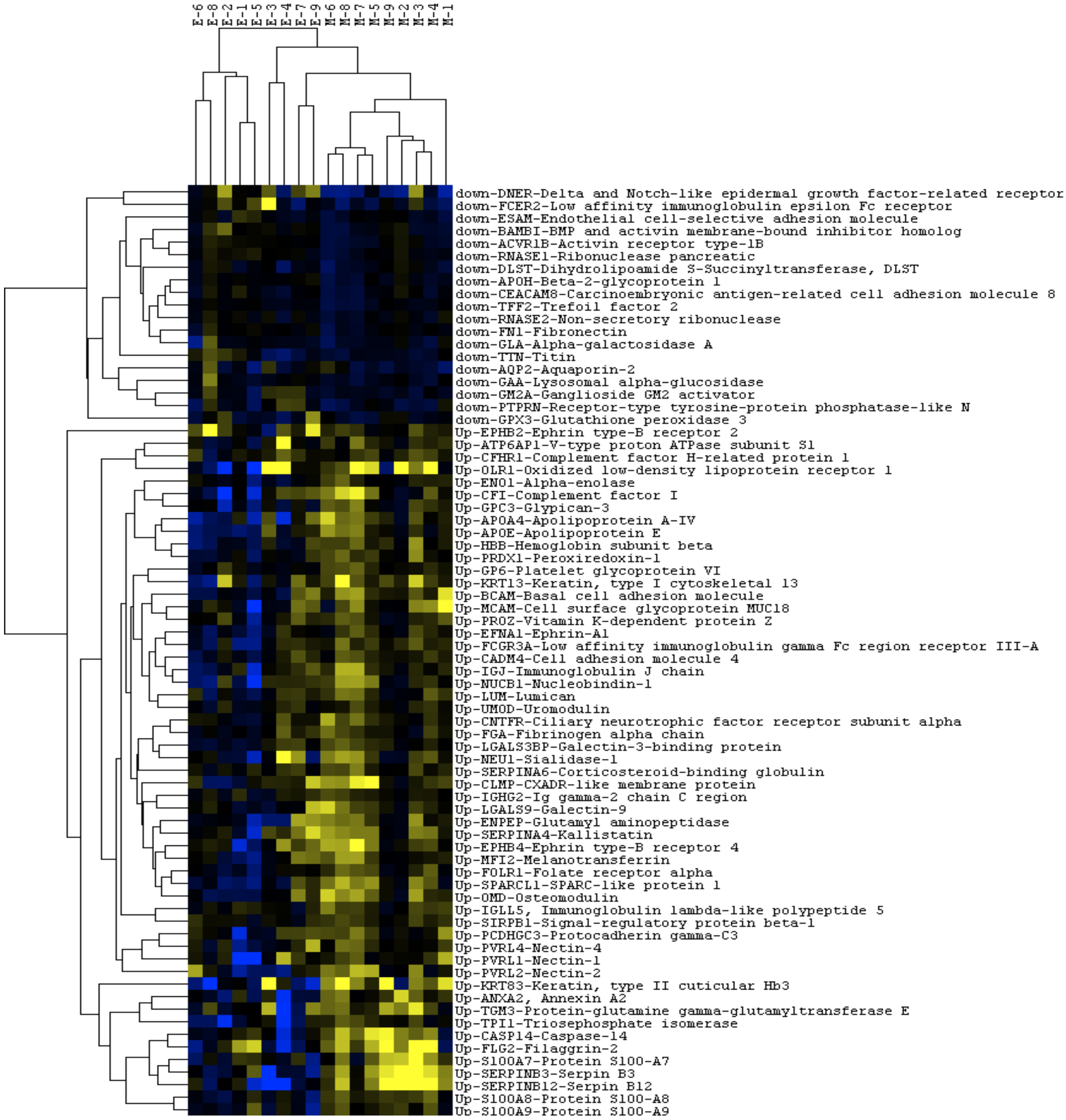

Supplementary Figure 6. Hierarchical cluster of protein ratios with full cluster.
Depicted are all (yellow) and downregulated (blue) proteins when clustering all proteins significant different between wet and dry nights in children with enuresis (n=84) (fold change (FC)>1.5 or <0.67, *P*<0.05). The evening values cluster to the left and are less homogeneous.
M (morning) = Wet / Dry ratio (FC) in nocturnal urine production from children with enuresis.
E (evening) = Wet / Dry ratio (FC) in urine samples collected before sleep from children with enuresis.
